# Harvesting the Promising Fruits of Genomics: Applying Genome Sequencing Technologies to Crop Breeding

**DOI:** 10.1371/journal.pbio.1001883

**Published:** 2014-06-10

**Authors:** Rajeev K. Varshney, Ryohei Terauchi, Susan R. McCouch

**Affiliations:** 1 International Crops Research Institute for the Semi-Arid Tropics (ICRISAT), Hyderabad, India; 2 The University of Western Australia (UWA), Crawley, Western Australia, Australia; 3 Iwate Biotechnology Research Center, Kitakami, Iwate, Japan; 4 Cornell University, Ithaca, New York, United States of America; The University of North Carolina at Chapel Hill, United States of America

## Abstract

Next generation sequencing (NGS) technologies are being used to generate whole genome sequences for a wide range of crop species. When combined with precise phenotyping methods, these technologies provide a powerful and rapid tool for identifying the genetic basis of agriculturally important traits and for predicting the breeding value of individuals in a plant breeding population. Here we summarize current trends and future prospects for utilizing NGS-based technologies to develop crops with improved trait performance and increase the efficiency of modern plant breeding. It is our hope that the application of NGS technologies to plant breeding will help us to meet the challenge of feeding a growing world population.

## Abstract 2

Rajeev Varshney, Ryohei Terauchi, and Susan McCouch summarize the current and future uses of next-generation sequencing technologies, both for developing crops with improved traits and for increasing the efficiency of modern plant breeding, as a step towards meeting the challenge of feeding a growing world population.

This article is part of the *PLOS Biology* Collection “The Promise of Plant Translational Research.”

## Introduction

In 2012, the world population exceeded 7 billion people and is expected to continue growing. To feed this growing population and meet rising expectations regarding food quality, food production must increase by an estimated 70% by 2050 [1]. Recent abrupt climatic changes [2] make stable food production even more difficult and put pressure on fragile environments. There is, therefore, an urgent need to accelerate crop breeding improvements and to implement new management strategies that together can achieve sustainable yield increases without further expanding farmland or damaging the environment [3].

To meet these challenges, scientists are developing new and more efficient breeding strategies that integrate genomic technologies and high throughput phenotyping to better utilize natural and induced genetic variation. Rapid developments in next generation sequencing (NGS) technologies over the last decade have opened up many new opportunities to explore the relationship between genotype and phenotype with greater resolution than ever before. As the cost of sequencing has decreased, breeders have begun to utilize NGS with increasing regularity to sequence large populations of plants, increasing the resolution of gene and quantitative trait locus (QTL) discovery and providing the basis for modeling complex genotype-phenotype relationships at the whole-genome level.

**Figure 1:**
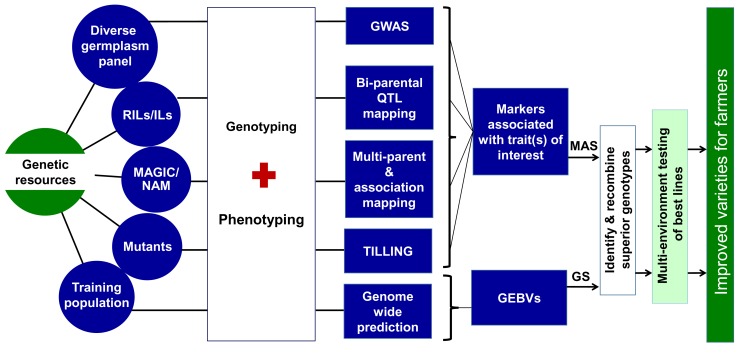
Role of NGS in genomics-assisted breeding. NGS occupies a critical position in a genomics-assisted breeding pipeline; it helps improve the speed and precision of trait mapping to identify genes and QTLs that are the targets of MAS, and it underlies the ability to calculate GEBVs based on genome-wide prediction that predict the breeding value of individuals in a breeding population using GS.

Specialized plant genetic stocks, such as bi-parental and multi-parent mapping populations, mutant populations, and immortalized collections of recombinant lines (Figure 1), have been generated to facilitate mapping and gene function analysis via association studies and QTL mapping (Box 1) in several crop species. Knowledge about the identity and map location of agriculturally important genes and QTL provides the basis for parental selection and marker-assisted selection (MAS) in plant breeding. Alternatively, genotypic and phenotypic datasets on training populations (TP; Box 1) can be used to develop models to predict the breeding value of lines in an approach called genomic selection (GS). We discuss both approaches later in this Essay.

**Box 1. Glossary**

**Bulked segregant analysis (BSA):** This approach identifies molecular markers associated with a trait of interest by genotyping DNA extracted from bulked samples of individuals at the trait's phenotypic extremes.

**Genome-wide association studies (GWAS):** These studies utilize collections of diverse, unrelated lines that are genotyped and phenotyped for traits of interest, and statistical associations are established between DNA polymorphisms and trait variation to identify genomic regions where genes governing traits of interest are located.

**Genotyping-by-sequencing (GBS):** A highly multiplexed genotyping system involving DNA digestion with different enzymes and the construction of a reduced representation library, which is sequenced using an NGS platform. It enables the detection of thousands of SNPs in large populations or collections of lines that can be used for mapping, genetic diversity analysis, and evolutionary studies.

**Marker-assisted back-crossing (MABC):** In this form of marker-assisted selection, a genomic locus (gene or QTL) associated with a desired trait is introduced into the genetic background of an elite breeding line through several generations of backcrossing.

**Multi-parent advanced generation inter-cross (MAGIC**): A type of multi-parent population developed from four to eight diverse founder lines, generated to increase the precision and resolution of QTL mapping because of the larger number of alleles and recombination events compared to bi-parental mapping populations.

**Nested association mapping (NAM)**: NAM combines advantages of linkage and association mapping and eliminates disadvantages of both; it takes into consideration recent and historical recombination events, facilitating high resolution mapping.

**Quantitative trait locus (QTL):** A genomic region encompassing one or more genes that accounts for a portion of the variation of a complex quantitative trait, identified by phenotyping and genotyping a segregating population followed by statistical analysis.

**Recombinant inbred line (RIL):** An immortal mapping population consisting of fixed (inbred) lines in which recombination events between chromosomes inherited from two inbred strains are preserved. RILs are generated by crossing two divergent parents followed by several generations of inbreeding to achieve homozygosity.

**Sequence-based mapping (SbM):** An approach requiring deep sequencing (5× to 8× genome coverage) of two DNA pools derived from individuals from the phenotypic extremes of a segregating population, to identify candidate genes associated with a phenotype of interest.

**Training population (TP)**: A genotyped and phenotyped reference breeding population used to develop a model to predict genomic-estimated estimate breeding values for Genomic Selection (GS).

**Whole genome re-sequencing (WGRS):** A strategy to sequence an individual genome where short sequence reads generated by NGS are aligned to a reference genome for the species, providing information on variants, mutations, structural variations, copy number variation, and rearrangements between and among individuals, based on comparison to the reference genome.

NGS technologies have been available for a number of years and are widely used for *de novo* sequencing, whole genome sequencing (WGS), whole genome re-sequencing (WGRS), genotyping by sequencing (GBS) (Box 1), and transcriptome and epigenetic analysis [4]. They are also used as the basis for developing fixed SNP genotyping arrays that typically consist of a set of well-distributed genic and non-genic SNPs. NGS strategies are now being improved by third generation sequencing (TGS) technologies (Box 2). TGS technologies can generate longer sequence reads in a shorter time and at even lower costs per instrument run. Collectively, NGS technologies have been used to sequence a range of plant species' genomes, mapping populations, and breeding lines. Their use has helped to broaden the plant research agenda over the last decade, and to shift from a focus on only one or two model species to a much wider range of plant species today. NGS technologies have also directly impacted the kind of plant science research that is undertaken in both the basic and translational research arenas.

**Box 2. Innovations in Sequencing Technologies**

In addition to classical Sanger sequencing methodology, a range of sequencing technologies have become available in recent years. These technologies are being used to sequence the genomes of a number of crops. Here we provide a brief update on these technologies and their use in sequencing the genomes of key plant species.

**Second/next generation sequencers (SGS/NGS)**

NGS technologies have enabled the whole genome sequencing (WGS) of several plant species and the re-sequencing of multiple genotypes [98],[99]. Two NGS approaches, *de novo* assembly and reference-based assembly, are employed for assembling short sequence reads into longer contigs. The sequencing of more plant genomes is expected as sequencing technologies become cheaper [4],[100].

**Third generation sequencing (TGS)**

The TGS approaches increase sequencing rates, throughput, and read lengths, ultimately decreasing sequencing costs and lowering the complexity of sample preparation. The current TGS technologies include: Ion Torrent's (Life Technologies) technology, a sequencer that uses semi-conductor technology to create a high-density array of micro-machined wells that carry out sequencing-by-synthesis, although it still requires PCR amplification of the DNA template and termination events, which limit read length to that of current NGS systems; Heliscope Single Molecule sequencer, which performs single molecule sequencing (SMS) [101],[102], the read lengths are 32 nucleotides long and no PCR amplification is required; Single-Molecule Real-Time (SMRT) sequencer performs sequencing by synthesis and overcomes many of the shortcomings of NGS [103], and produces maximum read lengths of 10,000 bp, enabling *de novo* assembly, however, the raw read error rates can be over 5%; Oxford Nanopore sequencing technology, which employs nanopore sequencing technology and a portable gene-sequencing device for use with GridION and MiniION single molecule sequencers, it offers 50–100 kb read length at 4% error rate.

Understanding the complex relationship between genotypic and phenotypic variation lies at the heart of the study of genetics and is also critically important to applications in plant breeding. Yet there is a considerable gap between the information that is available on model species about the genes and QTLs that underlie plant phenotypes and the integration of this information into applied plant improvement. In part, this gap is due to the geneticists' desire to simplify the genetic background (by using “wild-type” or “reference” populations that do not capture the complex genotype × genotype interactions in materials used by the breeding community), and minimize environmental “noise” (by using carefully controlled environments that do not capture the complexity of real-world environmental variation) to study the function of genes at a mechanistic level. The use of NGS for gene discovery in diverse species and populations, and as a foundation for large-scale modeling in both basic plant genetics and applied plant breeding, is helping to bridge the gap.

## Genomics-Assisted Breeding

Here we discuss two main types of genomics-assisted breeding [5]: (1) MAS and (2) GS. MAS, which includes marker-assisted back-crossing (MABC; Box 1), uses molecular markers that map within specific genes or QTLs known to be associated with target traits or phenotypes to select individuals that carry favorable alleles for traits of interest (and/or to discard those that do not). GS, on the other hand, uses all available marker data for a population as predictors of breeding value. Specifically, GS integrates marker data from a training population with phenotypic and, when available, pedigree data collected on the same population to generate a prediction model. The model outputs genomic estimated breeding values (GEBVs) for all genotyped individuals within a breeding population [6]. The GEBVs serves as a predictor of how well a plant will perform as a parent for crossing and generation advance in a breeding pipeline, based on the similarity of its genomic profile to other plants in the TP that are known to have performed well in the target environment(s). Before the prediction model can be applied to a breeding population, the accuracy of the model is generally tested using cross-validation on subsets of the training population. Once validated, the model can be applied to a breeding population where GEBVs are calculated for all lines for which genotypic information is available, and their phenotypic performance is predicted solely on the basis of that genotypic information.

The advantage of genomics-assisted breeding is that genotypic data obtained from a seed or seedling can be used to predict the phenotypic performance of mature individuals without the need for extensive phenotypic evaluation over years and environments. The use of genomics-assisted breeding, in both MAS and GS, allows for more selection cycles and greater genetic gain per unit of time. While some phenotyping is still advantageous to validate performance prior to further crossing or variety release, and in the case of GS, to maintain or increase the accuracy of prediction models as the breeding population evolves, extensive multi-location field trials become unnecessary in every generation.

Over the past several decades, as the process of selecting plants for breeding has shifted from an almost complete reliance on phenotyping to an increasing reliance on some level of genotyping-based methods, the number of markers used for selection has steadily increased. This has been made possible by NGS technologies that have augmented the speed, throughput, and cost effectiveness of genome-wide genotyping. Previously, marker data were expensive and laborious to generate, and marker-assisted breeding strategies were constrained by the number of markers that could efficiently be assayed. As a result, only markers in critical genomic regions were utilized to predict the presence or absence of agriculturally valuable traits. By contrast, the use of NGS technologies provides genome-wide marker coverage at a very low cost per data point, allowing us to assess the inheritance of the entire genome with nucleotide-level precision.

In the context of genomics-assisted breeding applications, both MAS and GS have benefited tremendously from NGS technologies. The resolution of most basic and translational studies is no longer limited by our ability to genotype large populations, but rather by the high cost and low throughput of phenotyping strategies for traits of interest and in environments relevant to plant breeding [7],[8]. As a result, breeders are looking for ways to leverage genotypic information, which is relatively fast, cheap, and easy to generate, to inform them about the phenotypic potential of their materials. Both MAS and GS are attempts to do that, and they each have different strengths and limitations. The utility of each depends on the genetic architecture and heritability of the trait(s) involved, the diversity of genetic backgrounds managed in the breeding program, the number of generations that a breeding population is removed from the original mapping or training population, and the overall organization and bioinformatics capabilities of the breeding program.

## Gene and QTL Discovery

The application of MAS in plant breeding is predicated on prior knowledge about major-effect genes and QTLs that serve as the targets of selection. NGS technologies have proven useful in identifying these loci in diverse populations. In the following section, we discuss various approaches to gene and QTL discovery where the use of NGS enhances the efficiency and resolution of the mapping process.

### Genome-Wide Association Studies

Genome-wide association studies (GWAS; Box 1) utilize association mapping, also known as linkage disequilibrium (LD) mapping, to map QTLs by taking advantage of historic LD to identify statistically significant phenotype-genotype associations (Figure 1). GWAS have been successfully performed in several crop plants, including maize [9]–[12], rice [13]–[15], wheat [16], soybean [17], sorghum [18], and foxtail millet [19]. The use of NGS in the context of GWAS makes it possible to genotype larger populations of plants with a higher density of markers than was previously possible, and this contributes directly to increased mapping resolution. With larger populations, more recombination breakpoints are identified, defining the position of candidate genes with higher precision. In parallel to developments in NGS technologies, specialized mapping populations have also been developed that significantly enhance the power and efficiency of GWAS. Nested association mapping (NAM; Box 1) populations were first developed for maize as a way of taking advantage of both historic and recent recombination events. This development was important to minimize the density of markers required by GWAS while taking advantage of the high allele richness, high mapping resolution, and high statistical power of association mapping [20],[21]. The NAM approach is similar in principle to the use of multi-parent advanced generation inter-cross (MAGIC; Box 1) populations, which are used to shuffle the genetic background among a set of diverse parental lines and increase recombination, and consequently the mapping resolution of QTL. Both types of population have been successfully developed and used to identify QTLs for a number of traits in diverse crop species [22]–[27].

### High Resolution Genetic Mapping and Candidate Gene Identification

NGS-based approaches, including sequencing-based mapping (SbM; Box 1), can be used in combination with bulked segregant analysis (BSA; Box 1) and modifications thereof to help speed the identification of candidate genes [28]. In BSA, DNA is extracted from plants at the extremes of the phenotypic distribution for a given trait, and samples from several plants at each of the extremes are pooled together and used to identify the genomic region(s) underlying the trait [29].

NGS-based approaches that involve whole genome sequencing can improve the power of BSA and are being widely used in many plant species today [30]–[37]. MutMap is a method based on WGRS of pooled DNA samples from the phenotypic extremes of a segregating population derived from a cross between a mutant of interest and the progenitor wild type line. Abe and colleagues [30] utilized this strategy to identify causal SNPs in a gene (*OsCAO1*) for the pale green leaf mutant in rice, and results were validated transgenically. In a related study, MutMap-Gap, was used to identify a major gene responsible for blast resistance, *Pii*, in rice where the resistance trait was associated with the presence of a nucleotide-binding site-leucine rich repeat (NBS-LRR) gene in a gap, that is, a structurally variable genomic region, where the resistance gene was not present in the reference genome used for WGRS alignment [37]. A similar approach, known as QTL-Seq, involves WGRS on bulked DNA samples from the phenotypic extremes of a population of recombinant inbred lines (RILs) (Box 1) or F_2_ individuals derived from inter-varietal crosses. The QTL-Seq strategy was used to identify QTLs for seedling vigour and partial resistance to blast disease in rice [36]. The QTLs were validated on the basis of classical QTL mapping studies, but the population sizes used to make the bulks (20–50 individuals each) were not large enough to provide gene-level resolution.

In another example, Xu and colleagues [38] re-sequenced 246 RILs of soybean and evaluated the lines for root knot nematode (RKN, *Meloidogyne incognita*) resistance to identify the gene(s) underlying a QTL for RKN resistance. RKN disease is difficult to evaluate phenotypically, but can cause up to 90% loss of susceptible soybean cultivars [39]. Identifying the genomic region(s) associated with RKN resistance was useful for developing resistant genotypes. Compared to previous marker systems, NGS is very efficient for map-based gene discovery because it simultaneously performs SNP discovery, SNP validation, and SNP genotyping in a mapping or mutant population. The work by Xu and colleagues [38] illustrates how NGS can also help resolve issues related to genome duplication in a complex, palaeopolyploid species like soybean.

### TILLING/Eco-TILLING by Sequencing

Targeting-induced local lesions in genomes (TILLING) is a reverse genetics approach for the rapid discovery and mapping of induced causal mutation responsible for traits of interest (Figure 1). Eco-TILLING is a method that uses TILLING techniques to identify natural mutations in individuals [40]. TILLING populations have been developed for several crop plants, such as rice [41],[42], wheat [43],[44], sorghum [45], oat [46], Brassica [47], chickpea and pearl millet (http://www.icrisat.org/bt-gene-discovery.htm), and used to identify useful alleles. To identify rare mutations in rice and wheat, Tsai and colleagues [48] developed a new approach called “TILLING-by-Sequencing,” in which target genes were amplified from pooled templates representing 768 individuals per experiment and then sequenced using NGS technology, leading to the discovery of novel rare mutants. Eco-TILLING, has also been used to identify novel variants of flowering related genes in sugar beet accessions [49], variants for a fatty acid desaturase gene in an olive (*Olea europaea*) collection [50], a reduced height (*Rht*)-1 gene in wheat [51], a drought tolerance transcription factor in rice [52], and genes associated with salinity stress tolerance in rice [53]. In the future, we envision that the use of Eco-TILLING and related approaches will enrich the gene pools of many crop species by identifying useful variants that have only rarely been used in modern crop improvement programs.

## Marker-Assisted Selection as a Breeding Practice

The oldest and most widely used type of genomics-assisted breeding is MAS. Identifying a gene or genomic region (QTL) that is responsible for a trait of interest is only an initial step in using MAS in a crop improvement program. Once found, the next step is to introgress the identified gene or genomic region(s) into an adapted crop line(s) using markers to identify the offspring that carry the most favorable combination of alleles. Both genotyping arrays and NGS approaches have been successfully used to introgress target loci into elite varieties to improve performance [54].

Simply inherited traits commonly targeted for MAS include disease and insect resistance, abiotic stress tolerance, and grain quality. MAS is particularly valuable for introgressing recessive alleles, pyramiding genes with overlapping phenotypic effects, for traits that are not expressed until late in plant development and traits that are difficult or expensive to phenotype [55]. MAS is used for both inbred and hybrid variety development [56]–[63]. In some crops, traits that are expected to have complex inheritance have been associated with major-effect QTLs that can be immediately targeted for MAS. Examples include “grain yield” and “yield under drought” in rice [64]–[66], biomass accumulation in Triticale [67], and drought tolerance in chickpea [68].

### Choice of Genotyping Platform

Fixed SNP genotyping arrays may be preferred to NGS technologies if they can achieve higher throughput at a lower cost per sample, or if they are specially designed to target high value functional alleles for traits of interest if a breeding program lacks the informatics support that would be required to decipher NGS information in a timely way. The density of SNPs on an array is typically less than the SNPs assayed by NGS, but the selection of array-based SNPs can be optimized for particular breeding applications. The reliability, turn-around-time, ease of information retrieval, as well as the cost of a genotyping assay and relationship to the service provider(s) are critical to a breeder where decisions about which individuals to advance to the next generation rely on timely access to genotypic information.

SNP-genotyping arrays, constructed from NGS datasets, have been developed and used to augment breeding efficiency in several crops, including maize (60 K SNPs [69]), rice (44 K SNPs [14]; 6 K SNPs [70]; 384 SNPs [71]), chickpea (2,068 SNPs [72]; 96 SNPs [73]), pigeonpea (1,616 SNPs [74]; 48 SNPs [73]), and groundnut (96 SNPs [75]).

### Use of MAS in Interspecific Populations

MAS and marker-assisted back-crossing (MABC) have been valuable for harnessing agriculturally valuable genes and QTLs from wild or unadapted genetic resources, particularly where the phenotype of a wild accession offers little or no insight about its potential value as a breeding parent [76],[77]. Prior to the advent of DNA markers, it was extremely cumbersome and inefficient to try to select for recombinant offspring from interspecific populations that carried the favorable wild allele(s) of interest because many unfavorable alleles that were also inherited from the wild donor typically masked the favorable phenotype. Genomics-assisted breeding has dramatically shifted the way breeders are able to work with unadapted genetic resources. Examples can be found in wheat [78]–[80], tomato [81], rice [13],[59],[64],[82],[83], maize [57],[84], barley [58], pigeon pea (http://goo.gl/zrdICo), chickpea [85], and foxtail millet [19].

NGS technology is vitally important as a tool for characterizing plant genetic resources globally. The vast majority of accessions found in the world's gene banks are currently poorly characterized and as a result, rarely used. An international effort is underway to take advantage of the low cost and high throughput of NGS, in combination with appropriate databasing of information, large-scale phenotyping, and population development, to help characterize gene bank materials and provide a rational basis for their utilization [86].

### Overcoming Linkage Drag

Breeders using MAS to introgress a favorable QTL allele from a wild or unadapted donor parent into an elite, adapted line often encounter the problem of linkage drag. The transfer of a large QTL region from a donor plant into a divergent breeding line may introduce undesirable phenotypic effects owing to the presence of linked genes in the introgressed QTL region. These linked genes often have nothing to do with the target trait but can make the new line unacceptable. NGS is vital for quickly identifying the individuals that carry critical recombination breakpoints that break the linkage drag. In one example, NGS was used to identify the recombinants to break linkage between a favorable allele conferring rice blast disease resistance and a deleterious gene affecting grain quality [87] and in another between a favorable allele conferring drought tolerance in rice and an unfavorable allele for tall plant stature [66]. Because the landraces that served as the breeding donors carried the favorable and the unfavorable alleles in coupling, it took a concentrated effort and deep sequencing within the target region on a large segregating population to identify a recombinant individual in which the linkage had been broken. In such cases, if the causal gene(s) and/or functional polymorphism(s) for the favorable and/or the deleterious trait(s) are known, the breeder can use that information to guide the selection of individuals that carry key recombination events to minimize the effect of linkage drag. Once a recombinant individual is identified, it becomes immediately useful as a donor in breeding and may serve to introduce new genetic variation into a breeding pipeline. In the case of Fukuoka and colleagues [87], the gene conferring blast disease resistance had not been used in breeding because previous attempts to introgress the resistance had been plagued by the poor grain quality trait. Thus, NGS can be extremely helpful to identify the recombinants in breaking linkage drag and liberating new forms of genetic variation for use in breeding.

## Genomic Selection as a Breeding Practice

As we have already discussed, GS does not depend on prior knowledge about a few, large-effect genes or QTL, and was not feasible prior to the development of genotyping technologies that provided high throughput, low-cost, genome-wide marker coverage. GS was originally developed for use in livestock breeding [6],[88], and is currently being applied to a wide range of crops [89]–[95]. The efficiency with which superior lines can be predicted through GS depends upon the genetic relationship between the training population and the breeding population, the number of generations that separate them, the type and number of markers used, the accuracy of the phenotyping, and the heritability of the trait(s) [90],[91],[96]. Where there is significant population structure within breeding populations of wheat and maize, pedigree information has been found to account for a large proportion of the prediction accuracy [89]. Genome-wide marker information can increase the prediction accuracy of the models, particularly where the trait is governed by many genes of small effect that are widely distributed throughout the genome. However, in many inbreeding species and wherever a trait of interest is governed by a few genes of large effect, specific information about SNPs within or near the target genes can enhance model accuracy and the value of GS.

### Combining Marker-Assisted and Genomic Selection

Despite the obvious differences between these two approaches to genomics-assisted breeding, there is much to be gained by combining the strengths of both approaches in the future. As information becomes increasingly available about which genes and alleles contribute to phenotypic variation in important breeding populations, greater weight can be given to specific polymorphisms that map within or very near to major-effect genes in GS models, which otherwise do a good job of tracking genes of small effect. Thus both approaches are critical as the plant breeding community seeks to enhance the productivity and sustainability of crop production in the face of climate change and increasing human demand.

## Perspectives

The development of improved breeding lines for commercial crop cultivation has traditionally been a time consuming and expensive task. With the deployment of genomics-assisted breeding, the generation of such lines is destined to become easier and faster, if also more expensive in the short term. To meet the demands of the human population and increasing volatility of the climate, we must accelerate the pace of our current breeding practices and apply genomics-based selection approaches.

Selection based on NGS allows marker discovery, marker validation, and genotyping itself to occur simultaneously, as we have discussed (Figure 1). The trend for sequence-based genotyping to replace the use of fixed marker arrays seems realistic, particularly as the cost of sequencing continues to fall, and is already happening for diploid crops with relatively small genome sizes (≤1 GB), such as rice (389 Mb), chickpea (738 Mb), sorghum (818 Mb), and pigeonpea (833 Mb). For polyploids and crops with larger genomes (e.g., bread wheat, a hexaploid with a 17 Gb genome), fixed SNP arrays will continue to be useful, particularly where they assay gene-specific or genome-specific markers that facilitate accurate mapping. Nonetheless, it is likely that NGS-generated data, including the many forms of GBS, will become the way of the future.

Currently, phenotyping is a major operational bottleneck that limits the power and resolution of many kinds of genetic analysis. We recognize the urgent need for high-throughput, cost-effective, and precise phenotyping methodologies that will undoubtedly involve digital image capture, remote sensing, and many new forms of information and communication technologies. To cope with the deluge of data generated from NGS and more automated phenotyping platforms, we need efficient data analysis and decision support tools to help breeders utilize that data in real time to select superior lines for crossing. We also need a massive reorganization of the way young plant scientists are trained [97], the way breeding programs are organized, and data are shared. We must integrate training across scientific fields, including genetics, plant breeding, computer science, mathematics, engineering, biometrics and bioinformatics, and to evolve new forms of communication and professional organization, so that genomics-assisted breeding can achieve its potential.

Finally, we need to provide suitable cultivars to farmers in a timely manner. While NGS-based approaches are helping improve the efficiency of breeding crops adapted to specific environments, we simultaneously need to provide farmers with information about the availability of new varieties about crop management systems and marketing opportunities. It is critically important that the efforts of the plant breeding community be fully integrated into the entire value chain so they can be rapidly and effectively deployed in farmers' fields, and so the fruits of genomics can ultimately reach the people they are intended to benefit.

## Abbreviations

GEBV: genomic estimated breeding value
GS: genomic selection
GWAS: genome-wide association studies
MAS: marker-assisted selection
NGS: next generation sequencing
QTL: quantitative trait locus
TGS: third generation sequencing
WGRS: whole genome re-sequencing
